# Effectiveness comparisons of acupuncture treatments for vascular dementia: A protocol for systematic review and network meta-analysis

**DOI:** 10.1097/MD.0000000000024079

**Published:** 2021-01-15

**Authors:** Xiuju Guan, Lijuan Zhang, Xinqin Li, Hanru Hou, Shuyue Bi, Kangfeng Wang

**Affiliations:** a First College of Clinical Medicine, Shandong University of Traditional Chinese Medicine; b Clinical Education Management Division, Affiliated Hospital of Shandong University of Traditional Chinese Medicine; c Shandong Vocational College of Special Education; d College of Traditional Chinese Medicine, Shandong University of Traditional Chinese Medicine; e Department of Neurology, Affiliated Hospital of Shandong University of Traditional Chinese Medicine, Jinan, Shandong Province.

**Keywords:** acupuncture, network meta-analysis, protocol, vascular dementia

## Abstract

**Background:**

Vascular dementia (VD) is the second most common form of dementia in the world. Acupuncture therapy has been widely used in clinical treatment. Based on the available evidence, we will rank different acupuncture therapy to determine the most effective acupuncture therapy.

**Methods:**

We will search the following database, including PubMed, Embase, Cochrane, Web of Science, China National Knowledge Infrastructure, Wanfang Database, Chinese Biomedical Literature Database and Chinese Scientific Journals Database database, in order to collect randomized controlled trials on acupuncture in the treatment of VD. We will use Stata 14.2 and WinBUGS 1.4.3 software for Bayesian network meta-analysis and finally evaluated the level of evidence of the results.

**Results:**

This study will compare and rank the effectiveness of acupuncture in the treatment of vascular dementia. Outcome indicators included Alzheimer Disease Assessment Scale-Cognitive section and Mini-mental State Examination, Activity of Daily Living, Blessed dementia scale, Hastgawa Dementia Scale, and adverse events.

**Conclusion:**

Our study will provide support for clinical practice.

**INPLASY registration number:**

INPLASY2020110088.

## 1 Introduction

Various pathogenic factors accumulate in cerebral blood vessels, affecting the important functions of cerebral blood vessels for oxygen supply, nutrient transport and nutrient signal transmission, leading to hypoxia and hypoperfusion in tissues, and inducing permanent tissue damage.^[1–3]^ Vascular dementia (VD) is the second leading cause of dementia after Alzheimer disease (AD), accounting for at least 20% of dementia cases.^[4,5]^ Relevant studies have confirmed that although the progression rate of vascular dementia is relatively slow compared with AD, its survival rate is lower than AD due to comorbidities, and the progress of effective treatment is more elusive than AD. Older age, education, and risk factors associated with vascular injury, such as hypertension, diabetes, hyperlipidemia, and hyperhomocysteine, all increase the risk of vascular dementia.^[6–8]^ The main treatment strategies for vascular dementia at present are reducing the incidence of vascular dementia by controlling the known risk factors of vascular dementia and preventing stroke recurrence through stroke management.^[9–11]^ Although drugs such as ganamine and cholinesterase inhibitors have shown significant but slight improvement in cognitive function in large randomized controlled trials, they have not been approved by regulatory authorities for use in patients with vascular dementia due to limited efficacy and possible side effects.^[12]^ Other drugs, such as calcium channel blocker Nimodipine and neurotrophic factor encephalin, have improved cognitive function, but they still need further clinical research and are not recommended to be widely used at present. Therefore, it is still an urgent problem to seek effective treatment methods.^[13,14]^

Acupuncture is a therapeutic method under the guidance of traditional Chinese medicine theory, which use specific therapeutic tools to perform acupuncture at specific body surface parts, namely acupoints, and achieves the therapeutic purpose by regulating the functions of viscera and meridians and promoting the operation of viscera, qi and blood. In recent years, there has been an increase in controlled clinical trials of acupuncture for VD, and acupuncture as an alternative medicine has been gradually accepted and widely used in clinical treatment.^[15]^ 555 Perng et al^[16]^ conducted a meta-analysis of clinical data from 2000 to 2016, and found that acupuncture, as an alternative therapy, is an appropriate and effective choice for patients with vascular dementia. Moreover, modern medical studies have shown that acupuncture can improve cognitive impairment through anti-oxidative stress, inhibiting inflammatory response, improving hippocampal synaptic plasticity and vascular function in model rats, promoting dopamine and receptor secretion, activating D1/D5Rs receptors and other mechanisms.^[17–20]^

Developed from traditional meta-analysis, network meta-analysis (NMA) can compare the efficacy of 3 or more interventions at the same time. The biggest advantage is to evaluate different interventions for the same disease ^[21]^ and rank them according to the effect of the outcome indicators, thus providing a basis for clinicians to make decisions. In this study, we will use NMA method for the first time to explore the effectiveness of different acupuncture methods in the treatment of vascular dementia, and rank them to work out the most priority plan for acupuncture treatment of vascular dementia.

## 2 Methods

### 2.1 Study Registration

The protocol for this systematic review was registered on INPLASY and the Registration number is INPLASY2020110088. (URL= https://inplasy.com/inplasy-2020-11-0088/).

### 2.2 Eligibility criteria

#### 2.2.1 Type of study

All randomized controlled clinical trials of acupuncture for vascular dementia published in Chinese or English will be included in the study, review, non-randomized studies, case reports, and retrospective studies will be excluded.

#### 2.2.2 Participants

All patients diagnosed with VD will be included in the study, and the diagnostic criteria will be based on the definition of vascular dementia formulated by NINDS/Airen Clinical Criteria for the Diagnosis of Vascular Dementia, CCDVD. Age, sex, course of disease, severity of illness, nationality, race, and duration will not be restricted.

#### 2.2.3 Interventions and comparators

The intervention measures of the experimental group included common acupuncture, scalp acupuncture, acupuncture, ear, electro acupuncture acupoint catgut embedding, used alone or combination of any 2 methods, or in combination with western medicine which should be the same as that of the control group. Laser acupuncture, bee venom acupuncture, acupoint application, bleeding and acupressure will be excluded. Acupuncture manipulation, frequency and duration will not be subject to special restrictions. The control group will use conventional western medicine or different acupuncture methods from the experimental group.

#### 2.2.4 Outcomes

The main outcomes are the vascular dementia Assessment Scale (VADAS-COG) and the Simple Mental State Examination (MMSE) scale and adverse events (rash, itching, pain, etc.).

The Secondary outcomes will include:

1. Activity of Daily Living (ADL)
2. Blessed dementia scale
3. Hastgawa Dementia Scale (HDS)

### 2.3 Search strategy

After systematically learning the method of literature retrieval, we determine the retrieval scope and strategy. We will search the following sources regardless of date, language, or publication status: PubMed, Embase, Cochrane, Web of Science, China National Knowledge Infrastructure, Wanfang Database, Chinese Biomedical Literature Database, Chinese Scientific Journals Database database, and ongoing clinical trials registered on the International Clinical Registration Platform. The search date for the system is built to October 30, 2020. The retrieval strategy will be constructed in the form of subject words combined with keywords, including “vascular dementia, acupuncture, scalp acupuncture, acupuncture ear, electroacupuncture, acupoint catgut embedding, randomized controlled trial,” etc. Take PubMed as an example, the search strategy is shown in Table 1.

**Table 1:** PubMed search strategy.

Number	Search terms
#1	vascular dementia[MeSH Terms]
#2	Dementias, Vascular[Title/Abstract] ORVascular Dementias[Title/Abstract] OR VascularDementia[Title/Abstract] OR Vascular Dementia, Acute Onset[Title/Abstract] OR Acute Onset Vascular Dementia[Title/Abstract] OR Subcortical Vascular Dementia[Title/Abstract] OR Dementia, Subcortical Vascular[Title/Abstract] OR Dementias, Subcortical Vascular[Title/Abstract] OR Subcortical Vascular Dementias[Title/Abstract] OR Vascular Dementia, Subcortical[Title/Abstract] OR Vascular Dementias, Subcortical[Title/Abstract]
#3	#1 OR #2
#4	Acupuncture[MeSH Terms] OR Pharmacopuncture[Title/Abstract]
#5	Acupuncture, Ear[Title/Abstract] OR (electroacupuncture[Title/Abstract] OR Scalp Acupuncture[Title/Abstract] OR acupoint catgut embedding[Title/Abstract]
#6	#4 OR #5
#7	randomized controlled trial[MeSH Terms]
#8	Clinical trials[MeSHTerms] OR randomly[Title/Abstract] OR placebo[Title/Abstract] OR randomized[Title/Abstract] OR trial[Title] OR randomized controlled trial[Publication Type] OR controlled clinical trial[Publication Type]
#9	#7OR#8
#10	humans[MeSH Terms]
#11	#3AND#6AND#9AND#10

### 2.4 Study selection and data extraction

**Figure 1:**
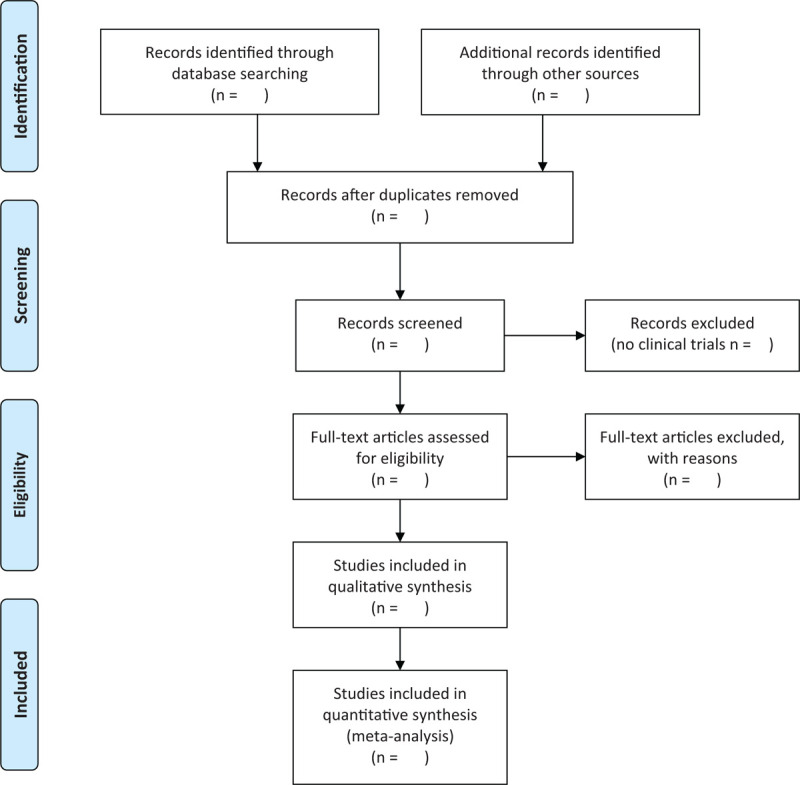
PRISMA flow chart.

We will import the literature extracted from the database into EndnoteX9 software, and delete duplicates through the preliminary screening of Endnote software and literature. By reading the titles and abstracts, we will eliminate the literatures that is repeatedly published and does not meet the inclusion criteria. Then, we will consult the full text, cross- checked, exclude the literature such as nonrandomized controlled trials and conference summaries, and make a record of the reasons for the deletion of the literature. Next, according to the pre-designed information extraction table, 2 reviewers will independently extract the data and cross-check it. In the event of a disagreement, the final decision will be made by a third reviewer. We will extract the information included basic information of the study, research methods, subjects, details of the intervention and control groups, outcome indicators and measurement methods, as well as the number of events and total number of dichotomous variables, and the total sample size, mean and standard deviation of the continuous variables. If information affecting NMA results is lacking in the literature, we will attempt to contact the original researchers for relevant information. The PRISMA flow chart of the selection process is shown in Figure 1.

### 2.5 Risk of bias assessment

Two researchers will independently assess the risk of bias in the included literature using the Cochrane Collaboration Risk of Bias Tool.^[22]^ Methods of random allocation generation, allocation concealment, participant blindness, outcome evaluator blindness, selective reporting, integrity of outcome data, and other sources of bias will be evaluated. Each field will be appropriately rated as high risk of bias and low risk of bias for uncertainty. The objection will be discussed by the 2 researchers to solve, if failed to agree on, will be settled by a third party to participate in.

### 2.6 Statistical analysis

#### 2.6.1 Pairwise meta-analysis

Two-by-two meta-analysis will be conducted using STATA15.0, and The odds ratio will be used as the effect indicator for the dichotomous variable, MD as the effect indicator for the continuous variable, and a 95% CI will be given for each effect index. Heterogeneity between studies was assessed by Cochrane Q and X^2^, and the size of heterogeneity was assessed by I^2^.

#### 2.6.2 Network meta-analysis

We will use Stata14.2 and WinBUGS1.4.3 software to perform Bayesian network meta-analysis and merge the data in the random effects model. We will map the evidence network to represent comparisons between studies, with the thickness of the edges representing the number of comparisons made, and the size of the points representing the number of participants. Bayesian network algorithm USES Markov chain Monte Carlo method for reasoning. Inconsistencies between direct and indirect comparisons will be assessed by the node-splitting method.^[23]^ By comparing the deviation information standards of each model, consistent and inconsistent models, fixed-effect models, and random-effect models were selected. The ranking of the effects of different acupuncture treatments will be presented by the surface under the cumulative ranking curve.

#### 2.6.3 Assessment of heterogeneity

If I^2^ < 50% and *P* > .1, we will use the random-effects model; If I^2^≥50% or *P* < .1, we will use the fixed-effects model.

### 2.7 Assessment of the similarity and transitivity

We will evaluate similarity and transitivity to produce credible and valid results. Since there is currently no accepted statistical test method, we will conduct an assessment based on clinical and methodological characteristics, and all factors influencing test results will be reported.

#### 2.7.1 Subgroup analysis and sensitivity analysis

If the heterogeneity is large, we will conduct a subgroup analysis of age, duration of treatment, course of VD and study quality to find the source of heterogeneity. We will conduct sensitivity analysis of outcome indicators and exclude references article by article. If the heterogeneity changes after excluding a certain reference, it indicates that the reference may be the source of heterogeneity. In addition, the reasons for heterogeneity can be explained by analyzing sample size, experimental design, outcome indicators, evaluation criteria, etc. If the heterogeneity remains unchanged after the exclusion of literature article by article, the results are relatively robust.

#### 2.7.2 Assessment of publication bias

We will study more than 10 articles and make funnel plot, and judge whether there is publication bias by observing whether the funnel plot is symmetrical.

#### 2.7.3 Grading the quality of evidence

We will use GRADE ratings to assess quality levels in 5 areas: risk of bias, indirectness, inconsistency, inaccuracy, and publication bias.^[24]^ The quality of evidence is divided into 4 levels: high, medium, low, and very low.^[25]^

#### 2.7.4 Ethics and dissemination

Ethical review is not required for this study. The results will eventually be published in peer-reviewed journals and disseminated electronically and in print.

## 3 Discussion

Vascular dementia is a neurodegenerative disease characterized by a decline in cognitive function that seriously affects daily life. The current treatment objectives are to improve patients’ cognitive level, improve their behavioral disorders and delay the progression of symptoms. There are still no drugs approved by regulators to treat vascular dementia. In recent years, acupuncture has been widely used in clinical treatment as an alternative therapy of Traditional Chinese medicine.^[26]^ In China, there are various acupuncture and moxibustion methods to improve vascular dementia, and clinicians are unable to determine the most suitable acupuncture and moxibustion methods for patients. Therefore, it is necessary to use NMA to analyze the comparative results of these acupuncture and moxibustion methods. NMA can compare the efficacy differences of multiple interventions to select the best treatment. This study is the first network meta-analysis of acupuncture in the treatment of vascular dementia. This meta-analysis will further confirm the efficacy of acupuncture in the treatment of vascular dementia and provide an evidence-based basis for clinical practitioners to select the best acupuncture method. There are still many limitations in our study. Firstly, frequency of acupuncture, duration of acupuncture treatment, manipulation of acupuncture, etc., will not be taken into account, which may lead to heterogeneity. Second, we will use research-level data rather than personal data, which may lead to bias in the results. However, our study will rank different acupuncture treatments and produce results that will benefit patients, clinicians, and policymakers.

## Author contributions

**Conceptualization:** Xiuju Guan, Kangfeng Wang; Lijuan Zhang.

**Formal analysis:** Hanru Hou, Shuyue Bi, Xinqin Li

**Methodology:** Lijuan Zhang, Xinqin Li.

**Project administration:** Lijuan Zhang.

**Software:** Xinqin Li, Hanru Hou, Shuyue Bi.

**Writing – original draft:** Xiuju Guan.

**Writing – review & editing:** Kangfeng Wang.

## Abbreviations

Abbreviations: AD = Alzheimer disease, NMA = network Meta-analysis, SUCRA = surface under the cumulative ranking curve, VD = Vascular dementia.
